# Data on the identification of isoprene and Styrene triblock copolymers with difunctional *t-*BuLi initiator

**DOI:** 10.1016/j.dib.2020.105272

**Published:** 2020-02-11

**Authors:** Pin-Chen Lee, Cheng-Chien Wang, Chuh-Yung Chen

**Affiliations:** a Department of Chemical Engineering, National Cheng Kung University, 701 Tainan, Taiwan, ROC; b Department of Chemical and Materials Engineering, Southern Taiwan University Science and Technology, 710 Tainan, Taiwan, ROC

**Keywords:** Isoprene, Styrene, Triblock copolymer, Gel Permeation Chromatography (GPC), 2D HSQC NMR

## Abstract

The data article refers to the paper “Synthesis of High-Vinyl Isoprene and Styrene Triblock Copolymers via Anionic Polymerization with Difunctional *t-*BuLi Initiator” [1]. Data presented here include the number average molecular weight (*M*_*n*_), the weight average molecular weight (*M*_*w*_), and polydispersity index (PDI) (*M*_*w*_/*M*_*n*_) of the triblock copolymers poly(styrene)*-b-*poly(isoprene)*-b-*poly(styrene) (PS*-b-*PI*-b-*PS, SIS) and poly(isoprene)*-b-*poly(styrene)*-b-*poly(isoprene) (PI*-b-*PS*-b-*PI, ISI). *M*_*n*_ of SIS and ISI were in the range of 208,000 to 274,000 (g/mol) and PDI of SIS and ISI are located at 1.18 to 1.2, respectively. The triblock copolymers were further identified with 2D HSQC NMR spectrum. Different vinyl content (1,2- and 3,4-addition units) of polyisoprene domains were characterized in the data.

**Table:** Specifications Table

Subject	Chemistry; Polymers and Plastics
Specific subject area	Anionic Living Polymerization
Type of data	Figures, Tables
How data were acquired	Gel Permeation Chromatography (GPC), Refractive Index (RI) detector (Viscotek VE 3580), 2D NMR HSQC (Bruker AVANCE III HD 600 MHz spectrometer)
Data format	Raw, analysed data
Parameters for data collection	Elution solvent, column, detector, and environment temperature for GPC analyst. Solvent, concentration for sample, and environment temperature for 2D HSQC NMR.
Description of data collection	GPC: Samples with different molecular weights and different vinyl groups were analysed by the RI detector after being injected into the instrument, and the computer collected data. 2D HSQC NMR: Samples with a fixed concentration were placed in the instrument, the data through Fourier transformed, and the file was output by the instrument software.
Data source location	Tainan, Taiwan, R.O.C.
Data accessibility	With the article
Related research article	Author's name: Pin-Chen Lee, Cheng-Chien Wang, and Chuh-Yung Chen Title: Synthesis of High-Vinyl Isoprene and Styrene Triblock Copolymers via Anionic Polymerization with Difunctional *t-*BuLi Initiator Journal: European Polymer Journal https://doi.org/10.1016/j.eurpolymj.2020.109476

**Table:** 

**Value of the Data** • Relevant data may be useful in the anionic polymerization of isoprene and styrene monomer, in particular for characteristic identification of isoprene with different structures. • Further understanding of the behaviour of styrene polymerization with different vinyl content of polyisoprene from this data. These data could be meaningful for understanding the interaction between the reaction of styrene with different viscosity of vinyl content of polyisoprene. • The identification methods for different vinyl content of polyisoprene are mostly ^1^H NMR identification. In addition to 2D HSQC NMR, which can confirm the characteristic peaks efficiently, it can also analyse the corresponding isoprene structure of the H-backbone. • Synthesis of SIS and ISI triblock copolymer with difunctional *t-*BuLi initiator may be the starting point for the further control the unique properties of block copolymers by using anionic polymerization.

## 1 Data description

**Fig. 1:**
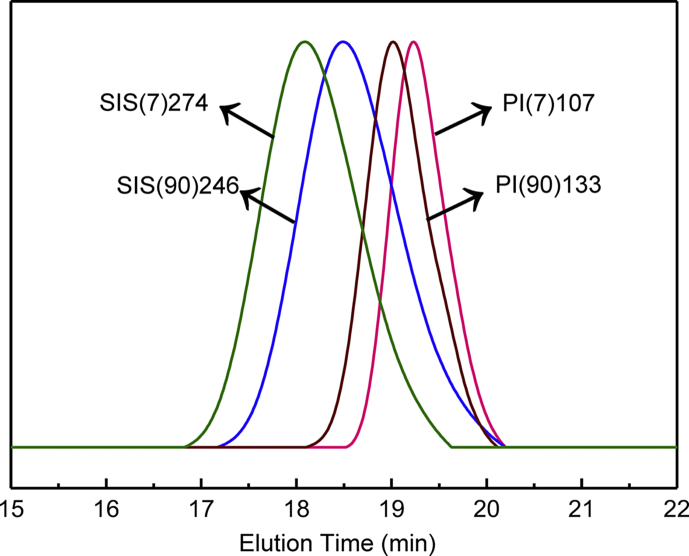
GPC traces of PI homopolymers and triblock copolymers SIS(7)274 and SIS(90)246.

The dataset contains raw data obtained the *M*_*n*_, *M*_*w*_, PDI (*M*_*w*_/*M*_*n*_) for the SIS and ISI triblock copolymers. Besides, the characteristic of the SIS and ISI triblock copolymers were also identified by 2D HSQC NMR. Fig. 1 shows the gel permeation chromatography (GPC) traces of PI homopolymers and SIS triblock copolymers. The synthesized samples were named as PI(V)M, where V is the percentage of vinyl groups (1,2- and 3,4-addition units) in the homopolymers and M is the number average molecular weight (*M*_*n*_) of the PI homopolymers in kDa. The *M*_*n*_ and PDI values of the PI homopolymers were shown in Table 1. The SIS triblock copolymers were synthesized following the polymerization of PI. The higher apparent molecular weight of SIS was the evidence of the structural transformation from PI to SIS. The SIS triblock copolymers had *M*_*n*_ values shown in Table 1 (see Fig.1).

**Fig. 2:**
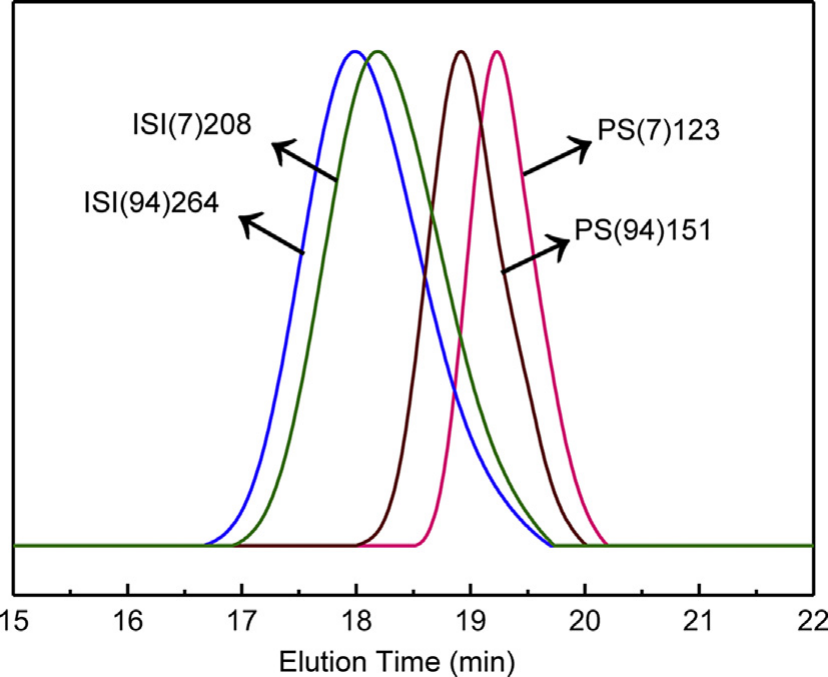
GPC traces of PS homopolymers and triblock copolymers ISI(7)208 and ISI(94)264.

**Table 1:** Characteristics and isomers of SIS triblock copolymers.

Sample	*M*_*n*_^a^ (kDa)		PDI^a^	PI isomers^b^			
				1,4 (%)	Vinyl group content(%)	3,4 (%)	1,2 (%)
PI(7)107	107.4	1.08		93.1	6.9	6.9	–
SIS(7)274	274.0	1.18		93.1	6.9	6.9	–
PI(90)133	132.7	1.12		10.1	89.9	64.2	25.7
SIS(90)246	246.4	1.20		10.1	89.9	64.2	25.7

a Calculated by GPC in THF at 30 °C.

b Calculated by ^1^H NMR.

On the other hand, the PS homopolymers had *M*_*n*_ values which were shown in Table 2. The synthesized samples were named as PS(V)M, where V is the percentage of vinyl groups in the triblock copolymers and M is the number average molecular weight (*M*_*n*_) of the PS homopolymers in kDa. The ISI triblock copolymers had *M*_*n*_ values which were shown in Table 2 (see Fig. 2).

**Table 2:** Characteristics and isomers of ISI triblock copolymers.

Sample	*M*_*n*_^a^ (kDa)	PDI^a^	PI Isomers^b^			
			1,4 (%)	Vinyl group content(%)	3,4 (%)	1,2 (%)
PS(7)123	122.6	1.19	93.4	6.6	6.6	–
ISI(7)208	208.0	1.21	93.4	6.6	6.6	–
PS(94)151	151.3	1.20	5.8	94.2	63.1	31.1
ISI(94)264	264.4	1.22	5.8	94.2	63.1	31.1

a Calculated by GPC in THF at 30 °C.

b Calculated by ^1^H NMR.

**Fig. 3:**
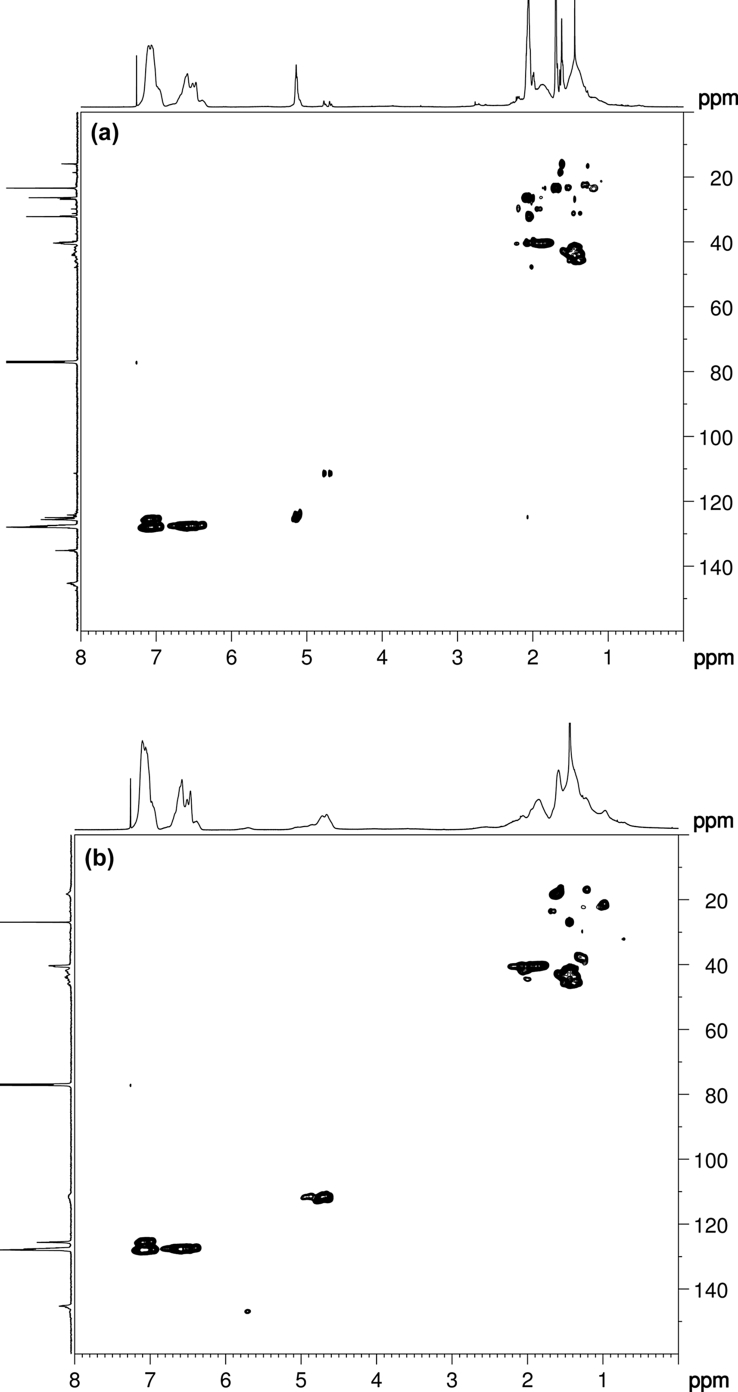
2D HSQC analysis of (a) SIS(7)274 and (b) SIS(90)246.

2D HSQC NMR spectroscopy experiments were performed to probe the relative positions of protons and their directly attached carbons in SIS triblock copolymers (Fig. 3). The main cross-signals in the aromatic regions of SIS(7)274 and SIS(90)246 were observed at δC = 127.2–127.9 ppm and δH = 6.9–7.2 ppm, respectively. The characteristic signals of 1,4-addition units in PI were observed at δC = 123.0–126.0 ppm and δH = 5.1 ppm. The chemical shift of δC = 111.8 ppm represented the isomerization structure of 3,4-addition units in PI, which correlated with δH = 4.6–4.75 ppm (Fig. 3(a)). Notably, the cross-signals in δC = 110.1–113.8 ppm and δH = 4.6–5.0 ppm represented the vinyl group isomerization structure (percentage of 1,2- and 3,4-addition units) in PI (Fig. 3(b)). Characteristic signals in the aliphatic region were observed at δC = 15.9–47.8 ppm and δH = 1.4–2.1 ppm. Furthermore, the *cis*- and *trans*-forms of 1,4-addition units could be distinguished using the 2D HSQC NMR spectrum (Fig. 3(a)).

## 2 Experimental design, materials, and methods

Isoprene (99%, Alfa Aesar) and styrene (SM) (>99%, Sigma-Aldrich) were purified from basic alumina column (99%, Macherey Nagel, Düren, Germany), dried over calcium hydride (>90%, Sigma-Aldrich), distilled under reduced pressure, and degassed by freeze−pump−thaw (three cycles). These materials were stored at 4 °C under argon. Cyclohexane (>99%, Duksan) and tetrahydrofuran (THF) (>99%, Duksan) were purified from calcium hydride, distilled, degassed by freeze−pump−thaw (three cycles), and stored at room temperature under argon. Methanol (99%, Duksan) and tert-butyllithium (t-BuLi) (1.9 M in Pentane, Rockwood Lithium) were used as received. For more detailed polymerization methods, please refer to our published paper [1].

### 2.1 Preparation of SIS and ISI triblock copolymer for GPC

The number average molecular weight (*M*_*n*_), weight average molecular weight (*M*_*w*_), and polydispersity index (PDI) (*M*_*w*_*/M*_*n*_) of the triblock copolymers were determined by gel permeation chromatography (GPC) with a refractive index (RI) detector (Viscotek VE 3580). The system was equipped with three linear columns (SHODEX KF-803L, KF-804L, and KF-805L) at 30 °C. Linear polystyrene (PS) was used for standard calibration, and THF (HPLC grade, Duksan) was the elution solvent at a flow rate of 1.0 mL/min. The injection concentration was 2.0 mg/mL and filtered before use.

### 2.2 Characteristic of SIS and ISI triblock copolymer with 2D HSQC

Nuclear magnetic resonance (NMR) spectroscopy measurements were performed using a Bruker AVANCE III HD 600 MHz spectrometer at 25 °C for 2D HSQC, using deuterated chloroform (CDCl_3_) as the solvent. 2D NMR HSQC determination was performed according to spectral widths of 3 kHz (5 ppm) for ^1^H and 15 kHz (100 ppm) for ^13^C. All NMR spectral analysis was conducted using Bruker Top Spin 4.0.4 software.

## References

[1] P.-C. Lee, C.-C. Wang, C.-Y. Chen, Synthesis of high-vinyl isoprene and styrene triblock copolymers via anionic polymerization with difunctional *t-*BuLi initiator, Eur. Polym. J. original research paper. 10.1016/j.eurpolymj.2020.109476.

